# Colour stability of Siloczest LSR 120 against Cosmesil M511 maxillofacial silicones under simulated environmental ageing conditions

**DOI:** 10.2340/biid.v13.45431

**Published:** 2026-02-06

**Authors:** Pavankumar Koralakunte Ravi, Anurag Jain, Sunitha Naveen Shamnur, Nandeeshwar Doddabasavaiah Basvapura

**Affiliations:** Department of Prosthodontics and Crown and Bridge, Bapuji Dental College and Hospital, Davangere, India

**Keywords:** colour stability, maxillofacial prosthesis, silicone elastomers, Siloczest LSR 120, spectrophotometry

## Abstract

**Background:**

Maxillofacial prostheses play a vital role in rehabilitating facial form and function, with long-term colour stability being a key determinant of patient satisfaction. These prostheses are routinely exposed to ultraviolet radiation, sweat, and cleaning agents, which may compromise their appearance over time.

**Aim:**

To compare the colour stability of two maxillofacial silicone elastomers, Siloczest LSR 120 and Cosmesil M511 under simulated ageing conditions replicating clinical exposures.

**Materials and methods:**

A total of 80 samples (40 per material) were fabricated and divided into four subgroups based on ageing conditions: sunlight, artificial sweat, immersion in neutral soap, and a combination of sunlight and sweat. Colour measurements were recorded before and after 6 months of ageing, except for neutral soap (30 hours) using the Commission Internationale de l’Eclairage Lab* ( CIE L*a*b*) system via a spectrophotometer, and ΔE values were calculated. Data were analysed using SPSS v28. Normality was assessed with Kolmogorov-Smirnov and Shapiro-Wilk tests. Independent *t*-test and one-way analysis of variance with Bonferroni correction were used for inter- and intra-group comparisons.

**Results:**

Both materials exhibited colour changes under all conditions. No statistically significant difference was observed between Siloczest LSR 120 and Cosmesil M511 under isolated sunlight or sweat exposure. However, Siloczest LSR 120 showed significantly higher discolouration under neutral soap (ΔE-1.48) and combined sunlight and sweat conditions (ΔE-6.68) against Cosmesil M511 (ΔE-0.68 & 3.82 respectively), (*p* < 0.05). The combination of sweat and sunlight caused the greatest colour degradation, particularly in Siloczest LSR 120.

**Conclusion:**

The results suggest that while Cosmesil M511 remains the more reliable option for long-term aesthetics, Silicozest LSR 120 demonstrates promising performance. Although its colour stability was comparatively poorer under neutral soap and combined exposure, the observed changes remained within clinically acceptable limits for darker skin tones (ΔE < 10.07), indicating that it can still be considered a viable material for clinical use in appropriate contexts.

## Introduction

The ability to look human is not merely a cosmetic concern; it is deeply tied to one’s identity and emotional well-being. Facial appearance plays a crucial role in how individuals perceive themselves and interact with others. Whether due to birth defects, trauma, or disease, the loss of facial structures can significantly impact a person’s confidence and social life [1].

Plastic and reconstructive surgery plays a pivotal role in the restoration process. However, in many cases, especially involving extensive disfigurement, surgery alone cannot provide a complete solution [2]. This is where maxillofacial prosthetics assumes critical importance. Prostheses are custom-designed external devices that aim to restore lost facial structures, such as the ear, nose, eye, or larger portions of the midface, with high fidelity. These devices serve not only a cosmetic function but also address deeper psychological and emotional needs by restoring a sense of normalcy and self-worth. Maxillofacial prostheses are especially vital in patients who are not ideal candidates for surgery or who prefer a non-invasive solution [3].

Among elastomeric materials, medical-grade silicones stand out for their unique combination of flexibility, softness, life-like appearance, and skin biocompatibility [4]. However, despite their widespread adoption, colour stability remains one of the most persistent and challenging issues in silicone prosthetics. A well-fabricated prosthesis can closely mimic natural skin tones at the time of delivery, but over time, its appearance may degrade due to discolouration. Environmental factors such as UV (Ultraviolet) radiation, temperature, humidity, air pollutants, and biological secretions like sweat and sebum contribute to pigment degradation and wear of silicone prostheses. Repeated cleaning, exposure to cosmetics, adhesives, and mishandling further accelerate ageing, leading to discolouration, hardening, surface roughness, and loss of life-like appearance. These changes compromise aesthetics, reduce patient confidence, and increase the need for replacements, adding financial burden and workload for prosthodontic services [5].

Silicone materials for maxillofacial prostheses are continually being developed to improve mechanical properties, biocompatibility, and aesthetic longevity. In addition, premium silicone materials are often expensive and may be difficult to access in resource-limited settings, creating financial and logistical challenges for patients and clinicians. Consequently, it is essential to scientifically evaluate newer silicone materials to ensure they meet clinical performance standards. Siloczest LSR 120 represents one of the newer additions to commercially available maxillofacial silicone materials. It is a platinum-based room temperature vulcanising (RTV) silicone elastomer designed for extraoral applications. Preliminary product information highlights favourable attributes, including high temperature resistance, flexibility, non-stick properties, and resistance to mould, bacteria, and moisture; however, these claims have not yet been validated through independent scientific studies [6].

Given the critical role of material performance in prosthesis longevity and patient satisfaction and knowing that changes in colour are among the most noticeable and distressing complications for patients, there is a need to assess whether newer materials perform comparably to established silicones. In addition, the clinical relevance of colour change must be interpreted using ΔE thresholds. For lighter and darker skin tones, ΔE values of up to approximately 3.35 and 10.07, respectively, have been suggested as clinically acceptable, as colour differences below these thresholds are generally not perceived during routine social interactions [7]. Therefore, evaluating whether a new material remains within this acceptability range under realistic conditions is important.

The environmental ageing conditions used in the present study such as exposure to sunlight (UV), perspiration, and neutral soap reflect common stressors encountered by patients using extraoral prostheses, especially in regions with high temperatures and humidity. These standardised conditions enable meaningful benchmarking of newer materials against established ones. Therefore, this study aimed to determine whether Silozest LSR 120, Chemzest Pvt Ltd, Chennai, India, marketed as a cost-effective alternative, performs comparably to an established material such as Cosmesil M511, South Wales, United Kingdom, under simulated extraoral ageing conditions, thereby providing evidence to support material selection and contributing to the existing scientific literature. The null hypothesis is that there will be no difference in colour stability between the two materials.

## Materials and methods

### Sample size determination

The sample size was estimated for the primary objective of comparing colour stability across eight groups. The calculation was based on an expected mean difference of 1.2 units (µA = 2.0 and µB = 3.2) with a standard deviation (SD) of 2, a Type I error (α) of 5%, and a power of 80% (β = 0.20) [8]. Considering the eight planned comparisons, the minimum required sample size was 71. To ensure adequate statistical power and equal distribution across groups, the sample size was rounded to 80, resulting in 10 specimens per group.

### Fabrication of the moulds

**Figure 1:**
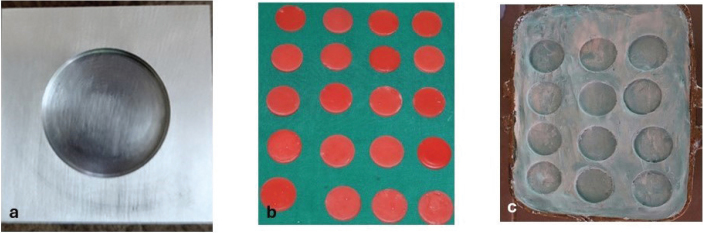
(a) Stainless steel die (30mm x 3mm), (b) Wax discs, (c) Custom made moulds.

The specimen dimensions were standardised at 30 mm in diameter and 3 mm in thickness. A customised stainless steel die of these dimensions served as the initial mould. Molten modelling wax, prepared according to the manufacturer’s instructions, was compressed into the die with a glass plate to ensure uniform thickness and smooth surfaces. Ten wax discs were prepared using this method, trimmed, and finished. These discs were invested in a custom-fabricated maxillofacial flask using Type III dental stone with a two-pour technique and separating medium. Once the stone had set, the wax patterns were removed, leaving precise disc-shaped moulds for fabrication of the silicone specimens (Figure 1).

### Preparation of the samples

#### Step 1: Manipulation ratios for silicone elastomers

The two commercially available maxillofacial silicones used in the study were:

1. Siloczest LSR 120 LSR 120 [Part A: Part B = 1:1] (Chemzest Pvt Ltd, Chennai, India)
2. M511 Maxillofacial Rubber [Part A: Part B = 10:1] (Cosmesil series material, Principality Medical Ltd, South Wales, United Kingdom)

The Siloczest LSR 120 silicone rubber consists of Part A and Part B mixed in a 1:1 ratio by weight (1 g of Part A and 1 g of Part B) [6]. In contrast, the Cosmesil M511 silicone rubber is mixed in a 10:1 ratio by weight (10 g of Part A and 1 g of Part B, approximately 20 drops) [9]. Mixing for both materials was performed on a white porcelain tile using a flexible maxillofacial silicone stainless steel spatula.

#### Step 2: Colour standardisation of the specimens

A standardised staining protocol was followed using intrinsic colour pigments. The elastomers and intrinsic colours were manipulated as per the manufacturer’s instructions to obtain the desired shade i.e. to match the Fitzpatrick IV to V range.

#### Step 3: Specimen fabrication

The prepared mould was thoroughly cleaned and dried. As per the manufacturer’s instructions, a neutral soap solution was used as a separating media and was applied evenly over the surface of the mould with the help of a small paint brush and allowed to dry. The final mixture was poured into the stainless-steel mould, and a glass plate was used to smoothen the surface. The mould was kept at room temperature for 24 hours before curing.

**Figure 2:**
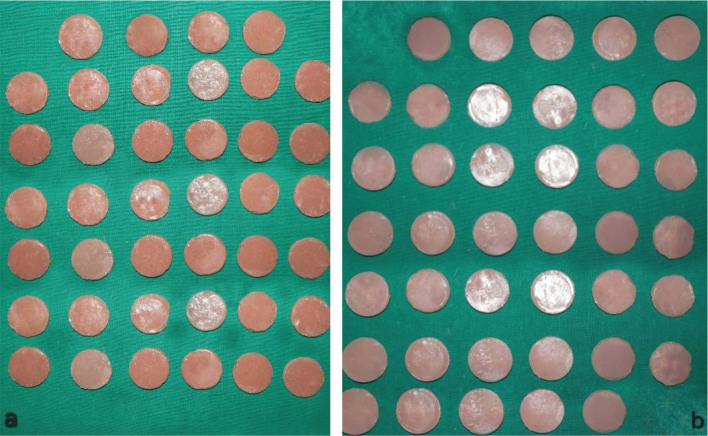
(a) Cosmesil M511 specimens, (b) Siloczest LSR 120 specimens.

After deflasking, specimens (Figure 2) were removed and inspected for surface irregularities, contaminants and internal defects. The flash and excess silicone was cut with a pair of sharp scissors. Any undesirable nodules were trimmed off using a sharp Bard Parker blade. The finishing of the specimens was done using silicone finishing burs (coarse and fine) followed by sand papering using 1200 grit sandpaper discs. The specimens were then thoroughly washed under running water and were cleaned in an ultrasonic cleaner with distilled water for a period of 15 minutes.

Specimens were accepted if diameter and thickness were within ±0.05 mm of target, showed no visible surface defects or air voids, and were non-tacky. Specimens showing surface defects, internal voids, warpage, incomplete cure, or colour/pigment heterogeneity were excluded and replaced.

### Measurement of colour value of the samples prior to extraoral ageing (Baseline readings)

After finishing and polishing, all specimens were dried, and colour measurements were obtained using a computer-controlled FRU Colour Spectrophotometer (WN700D/WN700S). The spectrophotometer was calibrated using standard white and black reference tiles in accordance with International Organization for Standardization (ISO) 7724-2:1984 and recalibrated after every 10 specimens. Measurements were recorded at the geometric centre of each sample under the Commission Internationale de l’Éclairage (CIE) Standard Illuminant D65 (ISO/CIE 11664-2:2022) using the CIE 1964 10° standard colourimetric observer as specified in ISO/CIE 11664-1:2019. Colour coordinates were computed using the CIE L*a*b* system. Three consecutive readings were taken for each specimen, and the mean L*, a*, and b* values were recorded.

### Grouping and exposure of the specimens to ageing conditions

**Figure 3:**
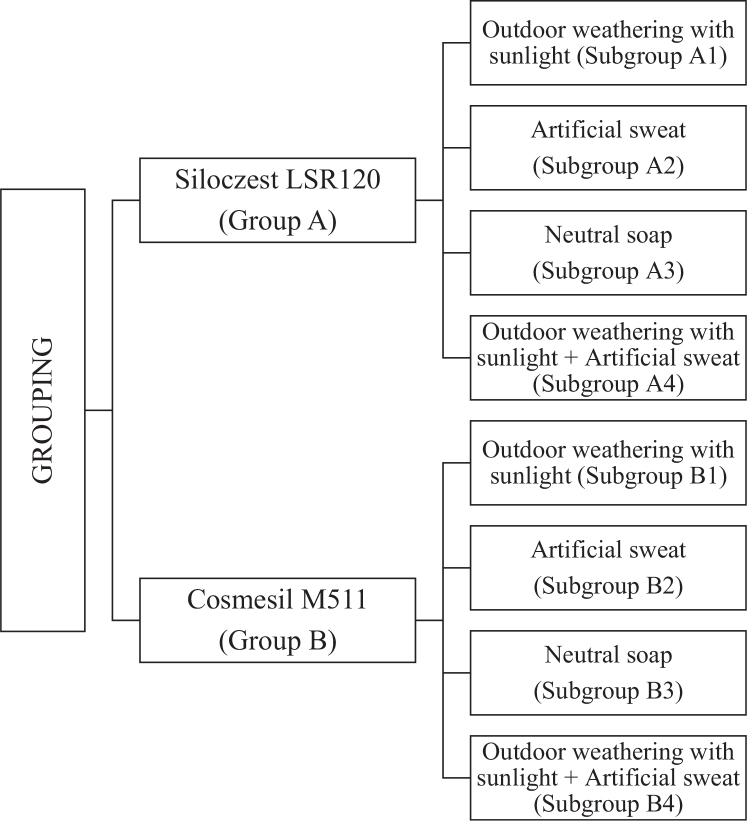
Sample grouping schema with corresponding subgroups.

A total of 80 specimens were fabricated and based on the materials used, they were then divided into Group A (Siloczest LSR 120) and Group B (Cosmesil M511) consisting of 40 samples each. The two main groups were further divided into four sub-groups (A1, A2, A3, A4 and B1, B2, B3, B4 respectively) containing 10 specimens each for testing colour stability under extraoral ageing conditions as follows (Figure 3).

- Subgroup A1, B1: Outdoor weathering with sunlight

Specimens were placed outdoors under sunlight in a petri dish inside a transparent glass chamber to protect it from rain and dust for a period of 6 months. During the study period (November 2024–April 2025), the region experienced typical tropical climate conditions, with an average daily temperature of approximately 26°C, relative humidity around 50–55%, a high UV index, and nearly 11 hours of daylight.

- Subgroup A2, B2: Artificial sweat

Specimens were immersed in the artificial sweat solution in airtight containers for 6 months and kept in an incubator at 37°C. Artificial sweat used was according to the ISO Specification No: 105-E04:87. The artificial sweat solution was replaced once per week to limit microbial growth, evaporation, and pH drift, while maintaining a feasible protocol for long-term immersion, acknowledging that no consensus standard currently exists for sweat solution renewal frequency in maxillofacial silicone studies.

- Subgroup A3, B3: Neutral soap

A neutral soap solution (Johnson & Johnson, VVF, Solan, India) was prepared by placing soap in 1 litre of distilled water. The samples were kept in continuous immersion inside airtight containers, protected from direct sunlight, and stored at an average room temperature of 26°C for 30 hours.

- Subgroup A4, B4: Combination of outdoor weathering and artificial sweat

Specimens were immersed in artificial sweat and simultaneously placed under outdoor sunlight for 6 months. The artificial sweat, contained in an airtight vessel, was kept inside a transparent glass chamber. Study specimens were fully immersed in this solution and exposed to direct sunlight. The artificial sweat solution was replaced weekly. Since the exposure was during the same period as that of subgroups A1, B1, they experienced identical climatic conditions, including comparable temperature, humidity, and natural sunlight exposure.

At the end of the exposure period, specimens were removed, ultrasonically cleaned for 10 minutes, rinsed under running water, dried, and subjected to colour measurement.

### Measurement of colour value after subjection to ageing conditions

Colour measurements of each specimen were performed using the FRU spectrophotometer. For each time point (baseline T0, after 30 hours of immersion T30h, and after 6 months of immersion T6m), three consecutive readings were taken at the geometric centre of the specimen, and the mean L*, a*, and b* values were recorded for each interval using the same standardisations as mentioned above.

Colour differences (ΔE*) between baseline (T0) and each ageing interval (T30h, T6m) were then calculated using the CIELAB formula:


$$∆E_{\mathrm{ab}}^{*}=\sqrt{{\left(∆L*\right)}^{2}+{\left(∆a*\right)}^{2}+{\left(∆b*\right)}^{2}}$$

where ΔL*, Δa*, and Δb* are the differences in L*, a*, and b* values between baseline (T0) and the respective post-immersion time point (T30h or T6m).

The repeatability of measurements was evaluated by computing the mean and SD of 10 repeated measurements of randomly selected specimens. The mean and SD estimated from the specimens for each subgroup was statistically analysed.

## Statistical analysis

Data analysis was conducted using the software SPSS (Version 28, IBM Corp., USA). Descriptive statistics, including mean, SD, minimum, and maximum values, were calculated for each group. Since the assumption of normality is a prerequisite for parametric tests, normality was assessed using the Kolmogorov-Smirnov and Shapiro-Wilk tests. A significance value (*p* < 0.05) was considered indicative of non-normality.

An Independent Student’s *t*-test was used for intergroup comparisons of mean ΔE values. Intragroup comparisons were performed using one-way analysis of variance (ANOVA). When statistically significant differences were observed, post hoc pairwise comparisons were conducted using the Bonferroni correction to determine specific group differences. A significance level of *p* < 0.05 was considered statistically significant, with Bonferroni correction applied to adjust for multiple comparisons.

## Results

The colour measurement values for all groups are summarised in Table 1.

**Table 1:** Mean, SD, minimum, and maximum values of ΔE to summarise the central tendency and variability within each group and subgroup.

Extraoral ageing conditions	Groups	*N*	Mean	SD	Min	Max
Outdoor weathering with sunlight	Group A	10	5.15	0.5	4.31	5.9
	Group B	10	5.22	1.05	4.23	7.83
Artificial sweat	Group A	10	3.39	1.07	1.88	4.9
	Group B	10	2.67	1.23	0.86	4.39
Neutral soap	Group A	10	1.48	0.17	1.14	1.72
	Group B	10	0.68	0.26	0.35	0.99
Combination of both outdoor weathering with sunlight and artificial sweat	Group A	10	6.68	0.91	5.61	8.54
	Group B	10	3.82	1.65	1.92	6.89

*N*: Number of samples; SD: Standard deviation; Min: Minimum; Max: Maximum.

As shown in Table 2, the Kolmogorov–Smirnov and Shapiro–Wilk tests were non-significant across all groups and conditions (*p* > 0.05), indicating that the assumption of normality was met; therefore, parametric tests were used for subsequent analyses.

**Table 2:** Tests of normality.

Extraoral ageing conditions	Groups	Kolmogorov-Smirnov^a^			Shapiro-Wilk		
		Statistic	df	Sig.	Statistic	df	Sig.
Outdoor weathering with sunlight	Group A	0.123	10	0.200*	0.962	10	0.812
	Group B	0.239	10	0.110	0.808	10	0.118
Artificial sweat	Group A	0.146	10	0.200*	0.941	10	0.565
	Group B	0.165	10	0.200*	0.930	10	0.447
Neutral soap	Group A	0.160	10	0.200*	0.946	10	0.618
	Group B	0.199	10	0.200*	0.874	10	0.111
Combination of both outdoor weathering with sunlight and artificial sweat	Group A	0.187	10	0.200*	0.901	10	0.227
	Group B	0.184	10	0.200*	0.927	10	0.419

* This is a lower bound of true significance. aLilliefors Significance Correction.

Inter-group comparisons of ΔE values between Groups A and B were performed using Student’s *t*-test under four extraoral ageing conditions (Table 3). No significant differences were observed following exposure to sunlight or artificial sweat alone (*p* > 0.05). In contrast, Group A exhibited significantly higher ΔE values than Group B after exposure to neutral soap and the combined ageing condition (*p* = 0.001), indicating greater susceptibility of Group A materials to colour degradation under specific environmental and chemical conditions.

**Table 3:** Inter-group comparison (Student’s t-test) of ΔE values under extraoral ageing conditions.

Extraoral ageing conditions	Groups	*N*	Mean	SD	*t*	*P* value
Outdoor weathering with sunlight	Group A	10	5.15	0.50	–0.176	0.862
	Group B	10	5.22	1.05		
Artificial sweat	Group A	10	3.39	1.07	1.409	0.176
	Group B	10	2.67	1.23		
Neutral soap	Group A	10	1.48	0.17	8.251	0.001*
	Group B	10	0.68	0.26		
Combination of both outdoor weathering with sunlight and artificial sweat	Group A	10	6.68	0.91	4.789	0.001*
	Group B	10	3.82	1.65		

* Statistical significance set at 0.05; *N*: Number of samples; SD: Standard deviation.

A one-way ANOVA was conducted to compare the colour change (ΔE) under four different exposure conditions: Outdoor weathering with sunlight, artificial sweat, neutral soap, and a combination of outdoor weathering with sunlight and artificial sweat within Group A and Group B, i.e. intra-group comparisons.

Tables 4 and 5 show significant differences in ΔE values across exposure conditions for Groups A and B, respectively (Group A: *F* = 89.21, *p* < 0.001; Group B: *F* = 27.22, *p* < 0.001), indicating a significant effect of exposure type on colour change. In Group A, the combined outdoor weathering and artificial sweat condition produced the highest ΔE (6.68 ± 0.91), followed by outdoor weathering with sunlight alone (5.15 ± 0.50), artificial sweat (3.39 ± 1.07), and neutral soap (1.48 ± 0.17), suggesting that combined environmental and chemical exposure had the greatest impact on colour stability. In Group B, outdoor weathering with sunlight resulted in the highest ΔE (5.22 ± 1.05), followed by combined outdoor weathering and artificial sweat (3.82 ± 1.65), artificial sweat alone (2.67 ± 1.23), and neutral soap (0.68 ± 0.26), indicating that exposure to sunlight had the greatest impact on colour stability. For both groups, exposure to neutral soap resulted in the lowest ΔE values, indicating minimal impact on colour stability.

**Table 4:** Intra-group comparison of ΔE values under different exposure conditions (One-way ANOVA) for Siloczest LSR 120.

Extraoral ageing conditions	*N*	Mean	SD	*F*	*P* value
Outdoor weathering with sunlight	10	5.15	0.5	89.21	0.001*
Artificial sweat	10	3.39	1.07		
Neutral soap	10	1.48	0.17		
Combination of both outdoor weathering with sunlight and artificial sweat	10	6.68	0.91		

* Statistical significance set at 0.05; *N*: Number of samples; SD: Standard deviation.

**Table 5:** Intra-group comparison of ΔE values under different exposure conditions (One-way ANOVA) for Cosmesil M511.

Extraoral ageing conditions	*N*	Mean	SD	*F*	*P* value
Outdoor weathering with sunlight	10	5.22	1.05	27.22	0.001*
Artificial sweat	10	2.67	1.23		
Neutral soap	10	0.68	0.26		
Combination of both outdoor weathering with sunlight and artificial sweat	10	3.82	1.65		

* Statistical significance set at 0.05; *N*: Number of samples; SD: Standard deviation.

Bonferroni post hoc analysis was conducted to identify pairwise differences among exposure conditions. For Group A (Table 6), all pairwise comparisons between exposure conditions were statistically significant (*p* < 0.001). Outdoor weathering caused significantly greater ΔE than artificial sweat (mean diff. = 1.76, *p* < 0.001) and neutral soap (mean diff. = 3.68, *p* < 0.001), while the combined exposure (outdoor weathering and artificial sweat) produced significantly higher ΔE than outdoor weathering alone (mean diff. = −1.53, *p* < 0.001) and artificial sweat (mean diff. = −3.29, *p* < 0.001). Neutral soap caused the least discolouration, with the largest difference observed between neutral soap and the combined exposure (mean diff. = −5.20, *p* < 0.001), indicating that combined environmental and chemical exposure resulted in the greatest colour change.

**Table 6:** Bonferroni post hoc comparisons of ΔE values for Siloczest LSR 120.

Intra-Group comparison	Mean diff.	Std. error	*t*	*p*	95% CI lower limit	95% CI upper limit
A1–A2	1.76	0.34	5.24	< 0.001*	0.8	2.72
A1–A3	3.68	0.34	10.93	< 0.001*	2.72	4.64
A1–A4	–1.53	0.34	–4.54	< 0.001*	–2.49	–0.57
A2–A3	1.91	0.34	5.69	< 0.001*	0.96	2.87
A2–A4	–3.29	0.34	–9.78	< 0.001*	–4.25	–2.33
A3–A4	–5.2	0.34	–15.48	< 0.001*	–6.16	–4.25

* Statistical significance set at 0.05; *t* – distribution.

In Group B (Table 7), outdoor weathering produced significantly greater ΔE than artificial sweat (mean diff. = 2.55, *p* < 0.001) and neutral soap (mean diff. = 4.54, *p* < 0.001) but did not differ significantly from the combined exposure (mean diff. = 1.40, *p* = 0.065). Artificial sweat caused more colour change than neutral soap (mean diff. = 1.99, *p* = 0.003) but did not differ significantly from the combined exposure (mean diff. = −1.15, *p* = 0.198). The combined exposure resulted in significantly higher ΔE only when compared with neutral soap (mean diff. = −3.14, *p* < 0.001). Overall, materials in Group B were most affected by exposure involving sunlight, either alone or in combination, while neutral soap consistently caused the least colour change.

**Table 7:** Bonferroni post hoc comparisons of ΔE values for Cosmesil M511.

Intra-Group comparison	Mean diff.	Std. error	*t*	*p*	95% CI lower limit	95% CI upper limit
B1–B2	2.55	0.52	4.9	< 00.001*	1.07	4.04
B1–B3	4.54	0.52	8.72	< 0.001*	3.06	6.03
B1–B4	1.4	0.52	2.69	0.065	–0.08	2.88
B2–B3	1.99	0.52	3.82	0.003*	0.5	3.47
B2–B4	–1.15	0.52	–2.22	0.198	–2.64	0.33
B3–B4	–3.14	0.52	–6.04	< 0.001*	–4.63	–1.66

* Statistical significance set at 0.05; *t* – distribution.

## Discussion

Silicone elastomers are preferred for maxillofacial prosthetics due to their flexibility, biocompatibility, and skin-like texture. They provide excellent marginal adaptation, tear resistance, ease of manipulation, and allow intrinsic and extrinsic pigmentation for accurate colour matching. Their chemical inertness and resistance to microbial growth support prolonged skin contact with minimal risk of allergy or infection, making them ideal for fabricating nasal, auricular, orbital, and other facial prostheses [10].

Colour stability is crucial for the long-term success and acceptance of maxillofacial silicone prostheses. Despite being biocompatible and life-like, silicones remain prone to degradation, with no material fully resisting colour changes under real-world conditions. Current silicones typically last 12–18 months, often showing physical and optical deterioration even sooner, making colour stability a key clinical challenge and an ongoing focus of material research [11, 12].

The aim of this study was to evaluate and compare the colour stability Siloczest LSR 120 with that of the established silicone, Cosmesil M511 under simulated extraoral ageing conditions: outdoor weathering with sunlight, artificial sweat, neutral soap, combination of both outdoor weathering with sunlight and artificial sweat. The results indicated that there was no statistically significant difference in colour change between the two groups when exposed to either outdoor weathering with sunlight or artificial sweat individually. However, statistically significant differences were observed when the samples were exposed to neutral soap solution and to the combination of outdoor weathering with sunlight and artificial sweat. Therefore, the null hypothesis was partially rejected.

Siloczest LSR 120 belongs to the LSR-1 series of liquid silicone rubbers and is a platinum-based RTV silicone. Its listed properties include high-temperature resistance, flexibility, and non-stick behaviour, with a hardness of 20 Shore A. It is low-viscosity and light-translucent, cures in 4–6 hours when mixed 1:1 by weight, is odourless, tasteless, hydrophobic, resistant to mould and bacteria, and does not require vacuum degassing. Specific details such as exact chemical composition, filler types, and proprietary additives have not been disclosed by the manufacturer [6].

Cosmesil M511 was chosen as the control material due to its widespread use in maxillofacial prosthetics. This platinum-cured RTV silicone is widely used clinically for its durability, biocompatibility, and excellent long-term colour stability. Its well-documented performance under various environmental conditions provides a reliable benchmark for evaluating other formulations like Siloczest LSR 120 [8, 13, 14].

The CIE Lab* spectrophotometric analysis was chosen as the preferred method for assessing colour stability due to its accuracy, reproducibility, and objectivity. The CIE Lab* system measures colour across three dimensions: L* (lightness), a* (red to green), and b* (yellow to blue), closely reflecting human visual perception of colour differences. This system allows for a standardised, quantitative evaluation of even minimal changes in shade, which is especially crucial in the clinical assessment of maxillofacial prostheses. Various studies assessing the colour stability of silicone prostheses have used this method to evaluate material performance over time and under different ageing conditions, as it reduces observer bias and provides consistent results across different samples and time points [8].

A 6-month conditioning period was selected to simulate 18–24 months of clinical service for silicone prostheses. Patients typically wear their prosthesis 8–12 hours daily, exposing it to about 6 hours of daylight and continuous contact with facial secretions, equating to roughly 4320 hours over 24 months. This was reproduced using accelerated ageing through outdoor weathering, simulated perspiration, sebum, and their combination. Additionally, a 30 hour immersion period in neutral soap and disinfectant solutions was used to mimic 12 months of routine cleaning, reflecting approximately 5 minutes of daily care, or 30 hours of cumulative exposure in a year [8].

Outdoor sunlight exposure significantly impacts the colour stability of maxillofacial silicone prostheses. Photo-oxidative degradation occurs through free radical formation, crosslinking, and chain scission. During the initial hours of UV exposure, some post-curing or additional crosslinking of un-crosslinked oligomers may occur; however, with prolonged UV/UVB exposure, photo-oxidative degradation becomes dominant. UV radiation penetrates the silicone and generates free radicals, leading to initial crosslinking and ultimately chain scission as high-energy photons break chemical bonds (e.g. C–H). This breakdown of long polymer chains into smaller fragments or cyclic oligomers alters the polymer’s refractive index, resulting in visible colour changes [5]. In the present study, both materials exhibited substantial discolouration after exposure to outdoor weathering, with Group A showing a ΔE of approximately 5.15 and Group B showing a ΔE of approximately 5.22. This aligns with previous findings: Hatamleh et al. reported notable colour change in high temperature vulcanised (HTV) silicone (ΔE ≈ 3.89) after 6 months of outdoor weathering, while Polyzois et al. observed progressive discolouration in RTV silicones over 1 year (ΔE 2.13–3.98). Similarly, Haug et al. found ΔE ≈ 3.86 after weathering and noted that intrinsic colourants may offer a protective effect by absorbing light and reducing degradation. Together, these studies highlight the susceptibility of maxillofacial silicones to environmental ageing [2, 15, 16].

Similarly, the permeation of sweat into the silicone matrix allows fatty acids to promote chain scission, while the acidic pH may facilitate crosslinking reactions, leading to further molecular alterations and discolouration over time [5]. In the present study, moderate colour changes were observed, with Group A showing a ΔE of approximately 3.39 and Group B showing a ΔE of approximately 2.67. These findings are consistent with the results of Hatamleh et al., who reported a ΔE of 4.51 ± 2.08 after 6 months of exposure to acidic perspiration, higher than the values observed in the present study, further underscoring the degradative effect of prolonged perspiration on maxillofacial silicones [2].

In contrast, neutral soap, with a mild pH of 7.0–7.6, does not significantly degrade the silicone polymer network; however, repeated mechanical cleaning can cause mild surface abrasion and pigment leaching, particularly when extrinsic colourants are present [17]. In the current study, minimal colour change was observed (Group A: ΔE ~1.48; Group B: ΔE ~0.68) indicating that neutral soap has the least impact on the colour stability of maxillofacial silicone materials. The relatively lower colour change observed may also be attributed to the absence of mechanical cleansing action, which represents a limitation of the study. Goaito et al. reported higher ΔE values (3.188 and 1.946) in two RTV silicones after neutral-soap exposure compared with the lower values in the present study, indicating greater colour change [10]. Similarly, Chamaria et al. observed a higher mean ΔE of 3.92 in RTV silicones exposed to antibacterial soap and noted that pigment loss from surface friction contributed to the significant difference between pigmented and non-pigmented groups [18].

When silicone prostheses are exposed simultaneously to sunlight and simulated acidic perspiration, these degradation mechanisms act synergistically, amplifying matrix disruption and pigment breakdown more than either factor alone, thereby accelerating colour changes. In the present study, this group showed the highest ΔE in both materials (Group A: 6.68, Group B: 3.82) indicating a compounded effect on colour alteration. Although no prior study directly evaluated the combined effects of outdoor sunlight and acidic perspiration, Hatamleh et al. assessed outdoor weathering with sebum exposure for 360 hours and reported a markedly higher ΔE of 10.78 ± 4.32 in HTV silicone (TechSil S25), compared with the much lower values in the present study, underscoring the severe discolouration that can occur under mixed ageing conditions [2].

In a recent study evaluating visual thresholds for maxillofacial silicones, the perceptibility (ΔE*ab) thresholds were reported as 0.8 for light skin and 2.63 for dark skin, while the acceptability thresholds were 3.35 and 10.07, respectively. These findings indicate that darker skin tones allow a wider margin of colour variation before the difference becomes noticeable or clinically unacceptable. As the present study used darker skin tones, the ΔE*ab values observed for both materials were well within the documented clinically acceptable limits [7].

It is difficult to extrapolate the results of this study to clinical conditions. However, the results of this study can give an insight into how different maxillofacial silicone elastomers may behave when exposed to different extraoral ageing conditions, thus affecting the clinician’s choice of material and the patient’s concern towards the prosthesis. The continuous ageing protocol imposes uninterrupted sweat contact and UV exposure, conditions not observed clinically because prostheses are routinely removed and cleaned. This likely exaggerates colour change; however, the high-stress model was intentionally employed as an accelerated ageing system to identify material failure thresholds and enable a standardised comparison of the chemical resistance of the two materials.

This study’s strengths include the use of clinically relevant individual ageing factors including sunlight, simulated sweat, and neutral soap applied through controlled and standardised protocols to allow meaningful comparison between materials. Evaluating two different silicone formulations and employing objective ΔE measurements ensured reliable quantification of colour changes. Additionally, confounding variables were minimised through uniform sample fabrication and tightly controlled spectrophotometric procedures.

However, this study has certain limitations. Colour stability was assessed only for intrinsic staining, without considering extrinsic staining or the effects of sealants and pigments. Silicone was mixed manually, and comparisons with vacuum mixing are needed. The CIE L*a*b* metric was used for consistency with existing maxillofacial literature, although it is less perceptually uniform than CIEDE2000. Finally, despite the material’s biomedical suitability, its long-term biocompatibility and clinical safety require further investigation.

## Conclusion

Cosmesil M511 demonstrated superior colour stability, particularly under combined environmental stressors, supporting its role as the more reliable material for long-term aesthetics. Silicozest LSR 120 showed greater discolouration under simultaneous sunlight and sweat exposure; however, all ΔE values remained within the clinically acceptable threshold for darker skin tones (<10.07). Although its performance was comparatively poorer in neutral soap and combined conditions, Silicozest LSR 120 still meets acceptable clinical standards, supporting its cautious use as an alternative material in appropriate contexts.

## Data Availability

No additional data are available. All relevant information is contained within the article.
